# Artificial Intelligence Versus Conventional Methods for NCCN Risk Stratification in Localized Prostate Cancer (2020–2025): A Systematic Review

**DOI:** 10.1155/proc/9923959

**Published:** 2026-07-21

**Authors:** Wael A. Hassan, Omar A. El Meligy, Iman M. Talaat

**Affiliations:** ^1^ Department of Clinical Sciences, College of Medicine, University of Sharjah, Sharjah, UAE, sharjah.ac.ae; ^2^ Research Institute of Medical and Health Sciences, University of Sharjah, Sharjah, UAE, sharjah.ac.ae; ^3^ Department of Pediatric Dentistry and Dental Public Health, Faculty of Dentistry, Alexandria University, Alexandria, Egypt, alexu.edu.eg; ^4^ Department of Pathology, Faculty of Medicine, Alexandria University, Alexandria, Egypt, alexu.edu.eg

**Keywords:** artificial intelligence, computer-assisted, image interpretation, machine learning, neural networks, prostatic carcinoma, risk assessment

## Abstract

**Background and Objective:**

Accurate risk stratification in localized prostate cancer is essential for guiding treatment decisions. Conventional National Comprehensive Cancer Network (NCCN) risk groups rely on prostate‐specific antigen (PSA), Gleason grade group, and clinical stage, while artificial intelligence (AI) methods, including radiomics, digital pathology, and multimodal prediction models, are increasingly being evaluated as alternatives or complements. This systematic review aims to assess studies published between 2020 and 2025 that directly compare AI‐based models with traditional NCCN risk stratification methods in localized prostate cancer.

**Methods:**

A systematic search of PubMed/MEDLINE, Scopus, and Cochrane Library (January 2020–September 2025) was conducted. Eligible studies focused on localized prostate cancer, used AI‐based models, compared them with NCCN risk stratification or its components, and reported outcomes such as the area under the receiver operating characteristic curve (AUC), sensitivity, specificity, calibration, or decision‐curve analysis. Case reports, nonhuman studies, and abstracts without full data were excluded. Title/abstract and full‐text screening was performed independently by two reviewers. Risk of bias was assessed using PROBAST, and reporting transparency was benchmarked against TRIPOD‐AI.

**Results:**

Out of 686 records, 100 duplicates were removed. Of the 586 screened, 95 were excluded. A total of 491 records mapped to NCCN components and compared AI with conventional methods; 43 full‐text studies met inclusion criteria. AI approaches combining MRI radiomics, PET imaging, and histopathology whole‐slide analysis consistently showed higher discrimination than NCCN models, especially for predicting adverse pathology and biochemical recurrence. However, calibration, external validation, and reporting quality were addressed inconsistently.

**Conclusions:**

AI‐based methods show promise in improving NCCN risk stratification for localized prostate cancer, delivering better prognostic accuracy than traditional approaches. However, variability in methods, limited external validation, and gaps in transparent reporting highlight the need for larger, multiinstitutional prospective studies before these methods can be widely adopted in clinical practice.

## 1. Introduction

Prostate cancer is one of the most common malignancies among men worldwide and a leading cause of cancer‐related mortality. Accurate risk stratification is fundamental for guiding clinical decision‐making, from active surveillance to radical therapy. The National Comprehensive Cancer Network (NCCN) risk classification remains the current standard, relying on serum prostate‐specific antigen (PSA) levels, Gleason score/grade group, percentage of positive biopsy cores, and clinical T stage. However, despite its broad adoption, NCCN stratification has limitations in accurately predicting individual patient outcomes, especially in intermediate‐risk disease, leading to both overtreatment of indolent cancers and undertreatment of aggressive disease [1].

Recent advances in artificial intelligence (AI), including machine learning (ML), radiomics, and deep learning applied to digital histopathology and imaging, have introduced novel opportunities to improve prostate cancer prognostication. AI models can analyze complex imaging features from MRI, PET, and whole‐slide images (WSI), as well as integrate multimodal clinical data, to predict adverse pathology and biochemical recurrence with greater discrimination than conventional methods [1–3]. Studies have demonstrated that AI‐assisted computational analysis, such as nondestructive 3D pathology and radiomics‐based nomograms, can outperform traditional 2D histology or NCCN grouping in predicting recurrence‐free survival [4, 5].

Between 2020 and 2025, there has been a significant rise in published studies directly comparing AI‐based prediction models with traditional NCCN risk stratification or its components. However, the results vary, with differences in validation methods, calibration, and reporting transparency. Methodological quality also remains an issue, requiring a structured critical appraisal using frameworks like PROBAST for bias risk assessment and TRIPOD‐AI for reporting standards [1, 4, 6–10].

Therefore, this systematic review synthesizes the available evidence from 2020–2025 on AI versus conventional NCCN‐based risk stratification in localized prostate cancer. Specifically, it evaluates whether AI approaches offer superior accuracy, calibration, and clinical utility compared to traditional risk grouping and highlights the strengths, limitations, and future directions of this rapidly evolving field.

In contemporary prostate cancer research, many AI models are designed to predict individual clinicopathologic variables, such as grade group, PSA level, clinical stage, or adverse pathological features, rather than composite NCCN risk categories directly. Because these parameters constitute the fundamental components of NCCN risk stratification and are used in clinical decision‐making, evaluating AI performance in predicting these elements provides a clinically meaningful and methodologically valid framework for assessing the potential of AI to refine or augment guideline‐based risk classification. Accordingly, this review considered studies that compared AI models with NCCN risk groups as well as those evaluating performance relative to NCCN components or established clinical nomograms derived from similar variables.

## 2. Methods

The systematic review was conducted in accordance with the Preferred Reporting Items for Systematic Reviews and Meta‐Analyses (PRISMA) guidelines [11] and was prospectively registered in PROSPERO (Systematic Review No. CRD420251159253).

### 2.1. Structured Research Question (PICO Framework)

A focused research question was formulated using the PICO framework. The population (P) included adult men with localized or locally advanced prostate cancer, with a preference for pretreatment cohorts. The index models (I) were AI or ML‐based models utilizing whole‐slide biopsy images, MRI or radiomics features, clinical parameters, or multimodal combinations aimed at predicting or assigning NCCN risk categories, individual components of NCCN categories (grade group, percentage/number of positive cores, PSA, and clinical T stage), or generating prognostic estimates directly compared with NCCN‐based stratification. The comparators (C) were conventional human‐based assessments such as pathologist grading, percent core involvement, radiologist staging, urologist assessment, NCCN risk grouping, or established clinical models/nomograms. The outcomes (O) included diagnostic or prognostic performance metrics such as discrimination (AUC), sensitivity, specificity, calibration, and clinical utility assessed by decision‐curve analysis.

### 2.2. Search Strategy

A comprehensive search was performed in PubMed/MEDLINE, Scopus, and Cochrane Library (January 2020 to September 2025). Reference lists of eligible studies were also screened (snowballing). Predefined keyword blocks were combined using Boolean operators (AND/OR) across four domains:

Artificial intelligence terms: “artificial intelligence” OR “AI” OR “machine learning” OR “deep learning” OR “neural network” OR “data mining” OR “algorithm” OR “automated” OR “computer‐aided” OR “multimodal artificial intelligence” OR “convolutional neural network” OR “predictive model”.

The AI‐related search terms were expanded to include additional contemporary model architectures, including transformer‐based models, to ensure comprehensive retrieval of recent studies.

Conventional methods: “conventional methods” OR “clinical parameters” OR “traditional” OR “clinical risk stratification” OR “NCCN risk” OR “National Comprehensive Cancer Network” OR “Gleason score” OR “PSA” OR “biopsy” OR “pathology”.

Prostate cancer scope: “localized prostate cancer” OR “prostate adenocarcinoma” OR “risk stratification” OR “prognostic group” OR “treatment decision” OR “active surveillance” OR “radical prostatectomy” OR “radiation therapy”.

The search strategy was further refined to include treatment‐related terms such as “androgen deprivation therapy” and “ADT” to ensure adequate coverage of studies evaluating treatment selection and outcomes relevant to NCCN‐based risk stratification.

Outcomes: “risk stratification” OR “risk classification” OR “prognostic model” OR “predictive model” OR “biochemical recurrence” OR “adverse pathology” OR “extracapsular extension” OR “lymph node metastasis” OR “organ‐confined”.

Outcome‐related search terms were expanded to include additional clinically relevant endpoints such as PSA failure, cancer‐specific survival, overall survival, and metastasis‐free survival, ensuring comprehensive identification of studies evaluating clinically meaningful outcomes.

Filters applied included English language, full‐text availability, and journals ranked Q1 or Q2 per SCImago.

### 2.3. Eligibility Criteria

Inclusion criteria were (1) localized prostate cancer population; (2) AI‐based models; (3) comparison with NCCN risk stratification or its components (Gleason grade group, PSA, clinical T stage, and percentage of positive cores); (4) outcomes including AUC, sensitivity, specificity, calibration, or decision‐curve analysis; and (5) original full‐text research in English.

Exclusion criteria were case reports, nonhuman studies, conference abstracts lacking full data, and studies not mapping to NCCN components or not comparing to conventional methods.

### 2.4. Study Types

Eligible designs included development/validation studies, external validation, prospective or retrospective cohorts, and reanalyzes of randomized controlled trial datasets.

### 2.5. Study Selection

**FIGURE 1:**
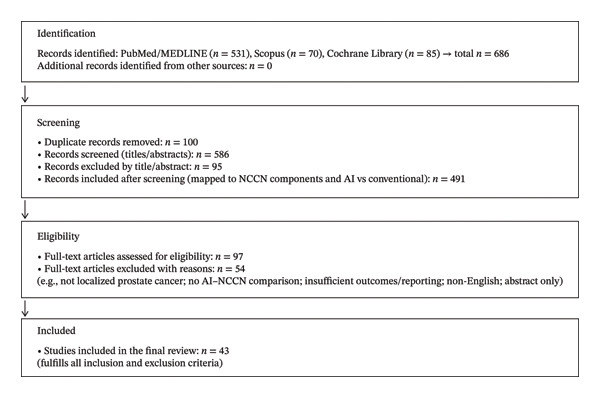
PRISMA flow diagram of study selection. The PRISMA flow diagram illustrates the systematic search and study selection process. From 686 initial records (PubMed/MEDLINE: 531; Scopus: 70; Cochrane: 85), 100 duplicates were removed, leaving 586 unique records for title/abstract screening. After excluding 95 records that did not meet inclusion criteria, 491 articles mapping to NCCN components were identified. Following full‐text assessment of 97 studies, 43 studies met all inclusion and exclusion criteria and were included in the final systematic review.

The initial screening of titles and abstracts (*n* = 686) was conducted independently by two reviewers. Studies meeting eligibility criteria were then subjected to full‐text examination, and reference lists of included articles were also screened to identify additional studies. Disagreements at any stage were resolved through discussion and, when necessary, consultation with a third reviewer to reach consensus. The final selection process is summarized in the PRISMA flow diagram (Figure 1).

### 2.6. Data Extraction

Two reviewers independently extracted data on study characteristics (author/year, country, design, number of patients, age, sample/treatment, study groups, objectives, and conclusions), as well as AI model type, comparator, outcomes, and NCCN components. Discrepancies were resolved by consensus, with a third reviewer consulted when necessary. Methodological uncertainties were clarified by contacting study authors.

### 2.7. Risk of Bias and Reporting Quality

The risk of bias of included studies was assessed using the Prediction Model Risk of Bias Assessment Tool (PROBAST), which evaluates participants, predictors, outcomes, and analysis domains [6]. Two reviewers performed the assessment independently, resolving discrepancies by consensus. Reporting quality was benchmarked against TRIPOD‐AI and CONSORT‐AI recommendations [10]. To improve clarity and adherence to systematic review reporting standards, the Results section presents descriptive findings and extracted outcomes only, while interpretation of findings, comparison with prior literature, and discussion of clinical implications are presented in the Discussion section.

## 3. Results

### 3.1. Search Results

A total of 686 records were retrieved from screened databases: PubMed/MEDLINE (*n* = 531), Scopus (*n* = 70), and Cochrane Library (*n* = 85). After removal of 100 duplicates, 586 unique titles and abstracts were screened. Of these, 491 articles mapped to NCCN components and compared AI with conventional methods. Following full‐text eligibility assessment (*n* = 97), 43 studies were included in the final review. The study selection process is illustrated in the PRISMA flow diagram (Figure 1).

### 3.2. Study Characteristics

The included studies (*n* = 43) were published between 2020 and 2025 and spanned multiple countries including China, USA, Canada, Argentina, Australia, the Netherlands, the United Kingdom, Sweden, Turkey, Japan, Germany, Austria, Italy, France, Switzerland, Cyprus, India, South Korea, South Africa, and Israel. Study designs ranged from retrospective cohorts and prospective observational studies to secondary analyses of phase III randomized controlled trials and large‐scale international multicenter validation studies. Sample sizes varied widely (from < 100 to > 100,000 participants).

AI methods included the following:

• Radiomics models based on biparametric or multiparametric MRI
• PSMA PET/CT‐based deep learning and radiomics classifiers
• Deep learning applied to digital histopathology (2D and 3D whole‐slide images)
• Multimodal AI models integrating pathology, imaging, and clinical data
• PSA‐based ML prediction models
• Convolutional neural networks (CNN) for biopsy and radical prostatectomy specimen analysis
• Active learning and domain‐agnostic deep learning systems
• ML models, including random forests, gradient boosting, support vector machines, neural networks, LASSO regression, and CART

Comparators were conventional NCCN risk stratification, its components (grade group, PSA, clinical T stage, and % of positive cores), or standard nomograms such as CAPRA‐S, the MSKCC nomogram, Partin tables, and the Roach formula. PI‐RADS scoring was also used as a comparator in imaging studies. Outcomes included discrimination (AUC and C‐index), sensitivity/specificity, calibration, decision‐curve analysis, concordance (Cohen’s kappa and quadratic weighted kappa), and survival analyses (biochemical recurrence‐free survival, metastasis‐free survival, and overall survival). Full study details are summarized in Table 1.

**TABLE 1:** Characteristics of included studies [13–19, 22–31, 36–42, 45, 46].

Author/year	Country	Study design	Number of patients	Age (years)	Sample/treatment	Study groups	Objective	Conclusion
Bjartell et al., 2025	Sweden	Prospective study	143	Median 64	Radical prostatectomy cohort	Training/validation/test cohorts	A multimodal AI bio marker was developed using clinical trial data from North American men with localized prostate cancer treated with definitive radiation, using biopsy digital pathology images and key clinical information (age, PSA, and T‐stage) to generate prog‐ n	MMAI significantly associated with outcomes (BCR: sHR 2.45, *P* < 0.001; AP: OR 4.85, *P* < 0.001)
Oliveira et al., 2024	USA	Retrospective comparative study across three surgical cohorts	777 patients		Radical prostatectomy cohort	Training/validation/test cohorts	Comparison of AI vs pathologist Gleason grading for metastatic outcome prediction	AI grading equivalent to pathologist grading (both C‐index 0.77)
Ma Q et al., 2025	China	Cohort study	116 (82 training, 34 testing)		Pelvic lymph‐node metastases in prostate cancer patients identified as candidates for extended pelvic lymph‐node dissection	Training/validation/test cohorts	PSMA PET/CT‐based multimodal deep learning model for accurate prediction of pelvic lymph‐node metastases in prostate cancer patients identified as candidates for extended pelvic lymph‐node dissection by preoperative nomograms	Superior performance (AUC 0.89) compared to existing nomograms
Öğülmüş FE et al., 2025	Turkey	Cohort study	229 patients (181 training, 48 testing)		Create a deep learning model to predict lymph‐node involvement in intermediate‐ to high‐risk prostate cancer patients	Training/validation/test cohorts	Retrospective AI model with reader study validation	AI outperformed radiation oncologists in reader study
Semwal H et al., 2025	USA	Retrospective machine learning using National Cancer Database	A total of 17358, 69292, 69292, and 17374 patients with PCa met inclusion criteria	Median 63		Training/validation/test cohorts	Seven ML models were trained to predict organ‐confined (OC) disease, extracapsular extension, seminal vesicle invasion (SVI), and lymph‐node involvement (LNI).	ML models better predicted pathologic stage relative to existing nomograms at predicting pathologic stage
Ueki et al., 2024	Japan	Retrospective study	625 patients	Median 63	Radical prostatectomy cohort			The light gradient‐boosting machine had the highest AUC of 0.924, followed by the random forest model with an AUC of 0.894
Liu et al., 2024	USA	Retrospective registry‐based ML model development with internal and external validation (FTR model)	118,236 with localized PCa: TD (TRD 44,621; TSD 41,500), IVD (IRD 4949; ISD 4621), EVD 22,545	Mean ± SD (training set): RT 67.3 ± 7.8 vs surgery 61.7 ± 7.3; similar in IVD/EVD (table values)	Localized PCa treated with RT or surgery in SEER	TRD/TSD, IRD/ISD, and EVD; “consistent” (treatment matched FTR) vs “inconsistent” (unmatched)	Build an AI model to identify which patients benefit more from RT vs surgery for cause‐specific survival	Consistent groups had higher survival than inconsistent; FTR accurately identifies which modality confers greater benefit in localized PCa
Adleman et al., 2025	USA/Canada	Retrospective cohort; AI segmentation (nnUNet) to derive PV and GTV; outcomes and toxicity analysis	187 brachytherapy pts: monotherapy 154; combination 33	Not reported in excerpt (age recorded as clinical factor)	LDR brachy monotherapy or brachy boost + EBRT (± ADT per practice)	Monotherapy vs combination subsets; test set (*n* = 187) and prior training cohort for the AI (external to this series)	Test whether AI‐GTV predicts BF/metastasis and AI‐PV predicts GU toxicity/IPSS resolution after brachytherapy	AI‐GTV associated with BF and metastasis; in monotherapy, AI‐PV associated with acute and late GU toxicity; findings may aid regimen selection
Dong et al., 2025	China (two tertiary hospitals, Wenzhou)	Multicenter retrospective radiomics study; training/internal/external validation cohorts	354 total: training 227, internal val 98, external val 29	Mean ± SD: training 68.38 ± 7.85, internal 69.00 ± 7.97, external 68.86 ± 7.05 years	Preop 18F‐PSMA‐1007 PET/CT radiomics; outcome persistent PSA (≥ 0.1 ng/mL at 6–8 weeks post‐RP)	Training/internal/external cohorts; models from intratumoral, peritumoral, and normal‐zone features, alone and combined with PSA	Develop zone‐based radiomics models (±PSA) to predict persistent PSA after RP	Multizone PSMA‐PET radiomics—particularly when combined with PSA—could improve prediction of persistent PSA after RP (internally and externally validated)
Medici et al., 2025	International, 11 centers in 5 countries (e.g., France, Italy, Switzerland, Germany, Cyprus; multicenter retrospective)	Retrospective multicenter cohort; mandatory PSMA‐PET staging; ML (LASSO⟶CART) for bRFS modeling	255 eligible (PSA ≤ 0.5 ng/mL, PSMA‐PET negative outside bed, no ADT)	NR	Salvage RT after RP; PSMA‐PET guided; doses per center; pelvic nodal RT per discretion	CART‐defined subgroups by pre‐SRT PSA, PSA‐DT, ISUP, PSMA‐PET positivity status, etc.	Identify prognostic factors and conditional interactions to refine bRFS prediction after SRT	Better bRFS with PSA < 0.2, PSMA‐PET negativity, longer PSA‐DT, favorable pathology; no dose‐dependent bRFS effect; CART isolated a high‐risk subset (PSA 0.21–0.50 ng/mL, PSA‐DT < 12 months, ISUP 3–5)
Maier et al., 2025	Germany (University Hospital Würzburg; LMU Munich) with collaborators (Cyprus)	Retrospective model development and evaluation on PSMA‐PET/CT; multiple CNN models tested (incl. post‐RP and intact prostate settings)	1145 patients; 1404 PSMA‐1007 PET/CT scans (2019–2023)	Mean 70.5 (range 44–90) years	PSMA‐1007 PET/CT; models A/B (whole and bladder‐focused), C (post‐RP), D (nonoperated)	Test whether DL can accurately detect local recurrence on PSMA‐PET/CT across clinical scenarios	Best model accuracy ∼77% with limited clinical utility; not yet sufficient for practice; code released to foster benchmarking	
Armstrong et al., 2025	USA (NRG Oncology Phase III trials; e.g., Duke, etc.)	Multimodal AI predictive biomarker (MMAI) trained on 6 randomized RT trials; prospective‐retrospective validation on RTOG 9202	Validation cohort *N* = 1192 (RTOG 9202)	NR	High‐risk localized/locally advanced PCa treated with RT + ST‐ADT (4 months) vs RT + LT‐ADT (28 months)	MMAI biomarker‐positive vs negative; treatment arms LT‐ADT vs ST‐ADT	Develop a biomarker to predict who benefits from LT‐ADT with RT (primary end point: distant metastasis)	LT‐ADT reduced DM overall and benefited biomarker‐positive men (sHR 0.55); no benefit in biomarker‐negative; ∼1/3 could avoid 24 extra months of ADT
Bergero MA et al., 2025	Argentina	Retrospective cohort study	1024 (training); 96 (validation)	Mean ∼60–61	Patients with localized PCa treated with robotic‐assisted radical prostatectomy (RALP)	BCR+ (476) vs BCR− (548); validation cohort (96)	To develop and validate ML‐based models (ANN, SVM, RF, XGBoost, DNN) for predicting biochemical recurrence (BCR) after RALP, and to compare them with CAPRA‐S	XGBoost achieved highest accuracy (AUC 0.91; validation AUC 0.89), outperforming CAPRA‐S. AI models provided better calibration and decision‐curve benefit, supporting improved individualized risk stratification
Wu SY et al., 2025	China	Multicenter retrospective study	666 (545 discovery, 121 test)	Median 67 (IQR 63–72)	Pretreatment bp‐MRI and clinicopathology data; radical prostatectomy	Discovery cohort (3 centers), test cohort (1 center)	To develop and validate bp‐MRI‐based radiomics and combined models for predicting biochemical recurrence (BCR), extracapsular extension (ECE), pelvic lymph‐node metastasis (PLNM), and Gleason grade group (GG).	The combined model (M3) using radiomics + clinical variables (without pathology results) showed superior predictive performance for BCR (AUC 0.884 in test cohort) compared to conventional models, offering a noninvasive tool for risk stratification and decision support
Parker CTA et al., 2025	UK, Switzerland, USA	Post hoc ancillary biomarker study of four Phase III RCTs (STAMPEDE)	3167 patients (1575 nonmetastatic, 1592 metastatic)	Median 68 (IQR 63–72)	Prostate biopsy samples; patients starting ADT ± docetaxel/abiraterone	SOC‐ADT, SOC‐ADT + docetaxel, SOC‐ADT + abiraterone	To externally validate a multimodal AI‐derived prognostic model (ArteraAI Prostate) for predicting prostate cancer‐specific mortality (PCSM) in advanced and high‐risk nonmetastatic PCa	The multimodal AI model was strongly prognostic of PCSM across metastatic and high‐risk nonmetastatic subgroups, improving risk stratification beyond conventional clinical variables
Ni X et al., 2025	China (Fudan University Shanghai Cancer Center)	Tumor marker prognostic study; development and external validation of ML‐based model	2716 patients (1160 SPARTAN, 1247 ARAMIS, after exclusions)	< 65, 65–74, ≥ 75; median not specified	Clinical trial data (SPARTAN, ARAMIS) including PSA, Gleason score, PSADT, HGB, prior treatments	Low‐, medium‐, and high‐risk groups based on ML‐derived risk score and risk factor count	To develop and validate a machine learning‐based prognostic model for predicting metastasis in nmCRPC patients	The LASSO + stepwise Cox model showed robust performance (C‐index ≈0.72–0.74; tAUC > 0.70), enabling effective stratification of nmCRPC patients and supporting individualized treatment planning
Zhang Z et al., 2025	China	Single‐center retrospective diagnostic study (imaging biomarker development/validation)	199 total (BPH‐only *n* = 106; BPH‐PCa *n* = 93); training *n* = 139, testing *n* = 60	NR	T2‐weighted MRI; deep learning segmentation (ProZonaNet) to derive prostate zonal volume ratio (pZVR = TZ/PZ)	BPH‐only vs BPH‐PCa; exploratory subset: low‐grade PCa (Gleason 3 + 3) vs BPH‐only	To develop and validate a deep learning‐derived prostate zonal volume‐based biomarker (pZVR) to distinguish BPH‐only from BPH‐PCa and potentially reduce unnecessary biopsies	ProZonaNet achieved superior segmentation; pZVR independently distinguished BPH‐PCa from BPH‐only and improved AUC when combined with age/PSA, indicating a promising noninvasive biomarker
Wu XH et al., 2024	China	Retrospective study	356 nonmetastatic PCa patients	Mean 69.2 ± 6.5	Preoperative mpMRI followed by radical prostatectomy (RP)	Training (*n* = 118), validation (*n* = 53), testing (*n* = 185)	To develop and validate a periprostatic fat MRI‐based radiomics nomogram predicting bRFS in nonmetastatic prostate cancer patients undergoing RP	Validated a radiomics‐clinical nomogram to predict bRFS in nonmetastatic PCa after RP
Tward JD et al., 2024	USA	Meta‐analysis of 8 NRG Phase III RCTs	9787 (2486 validation set)	Median 69 (IQR 63–73)	Radiation therapy ± ADT ± chemotherapy; digital histopathology evaluation	NCCN risk groups vs multimodal AI (MMAI) risk groups	To develop and validate a multimodal AI (MMAI) model combining digital histopathology and clinical data and compare its performance with NCCN risk stratification	MMAI improved stratification, reclassifying 42% of patients. It identified more patients as low‐risk with comparable outcomes and better stratified high‐risk patients, reducing over‐ and undertreatment
Shao Y et al., 2024	Canada (University of British Columbia, Vancouver)	Retrospective cohort, AI model development and validation	502 treatment‐naïve PCa patients	Mean 64.9 ± 7.5	Radical prostatectomy tissue microarrays (H&E and Ki‐67 stained)	CAPRA‐S (low ≤ 2, intermediate 3–5, high ≥ 6) vs AI‐derived CCHEK model	To evaluate whether a deep learning histopathology‐based risk stratification model (CCHEK) improves prediction of biochemical recurrence (BCR) and overall survival (OS) compared to CAPRA‐S	The CCHEK model outperformed CAPRA‐S, reclassifying patients more accurately for BCR and OS, suggesting utility for guiding adjuvant therapy decisions
Xie W et al., 2022	USA/China	Retrospective study	*n* = 497 (multiinstitutional dataset)	Median age not specified (∼60s)	Digitized H&E whole‐slide images; nondestructive 3D pathology reconstruction	Deep learning‐assisted 3D gland analysis vs conventional clinicopathologic models	To evaluate whether nondestructive 3D pathology with AI‐assisted gland analysis improves risk stratification in localized prostate cancer	Deep learning‐based 3D gland analysis improved prognostic accuracy compared to conventional 2D pathology, suggesting added value for NCCN risk stratification
Perera M et al., 2022	Australia, USA	Retrospective multicenter study (PLCO, PCPT, Australian cohort)	10,719	Median 61 (IQR 56.5–65.5); PCPT: 62; PLCO: 62; Australian cohort: 54	PSA data from PLCO, PCPT, and Australian screening cohort	PCPT trial (*n* = 5010), PLCO trial (*n* = 3786), Australian cohort (*n* = 1923)	To construct and validate a machine learning model using PSA kinetics and compare it with PSA and PSA velocity for PCa risk prediction	ML model significantly outperformed PSA and PSA velocity in predicting clinically significant prostate cancer (AUC 0.886 vs 0.807 and 0.627)
Chen S et al., 2022	China	Retrospective study (biopsy cohort, 2013–2021)	551	Mean not specified; subgrouped < 65 vs ≥ 65	Prostate biopsy population (TRUS‐guided, 10–12 cores)	Training dataset (*n* = 414), test dataset (*n* = 137)	To construct and validate ML‐based PCa prediction models (LR, DT, RF, SVM) compared to PSA	Multivariate LR (AUC 0.918) and SVM models performed best; ML methods significantly improved accuracy and net clinical benefit compared to PSA alone
Cysouw MCF et al., 2021	Netherlands	Prospective cohort study	76 patients	Mean 71 years (range 55–81)	(18F)DCFPyL PET/CT radiomics features; machine learning‐based analysis	Patients undergoing primary staging for high‐risk prostate cancer; ML models compared with NCCN risk group classification	To evaluate the added value of (18F)DCFPyL PET radiomics combined with machine learning for predicting biochemical recurrence‐free survival and compare performance with NCCN risk groups	ML radiomics models improved prognostic accuracy over NCCN risk groups alone, showing potential for enhanced individualized risk stratification in high‐risk prostate cancer
Yu S et al., 2021	China	Retrospective study (2016–2020)	688 (biopsy‐naïve men, tPSA ≤ 50 ng/mL)	Median 66 (no PCa) vs 70 (PCa)	mpMRI + TRUS‐guided prostate biopsy	Training cohort (480) and validation cohort (208)	To develop and compare ML models (ANN, SVM, RF, CART) incorporating mpMRI and PSA vs logistic regression for predicting PCa and CSPCa	SVM and RF models had similar diagnostic accuracy to LR while sparing more biopsies at 95% sensitivity; CART best calibration but lower accuracy; no method clearly superior
Perera M et al., 2021	Australia	Retrospective screening cohort study with ML model development and validation	*N* = 143 (screening population)	Median 65 years (range not specified)	PSA, free PSA, PSA derivatives; machine learning dense neural network model	PSA‐based ML model vs PSA and PSA derivatives (free PSA, free‐to‐total ratio)	To evaluate whether a PSA‐based machine learning model improves prostate cancer risk stratification in a screening population compared to conventional PSA parameters	The ML model outperformed PSA alone and its derivatives (AUC 0.72 vs 0.63), showing improved risk stratification and calibration, suggesting utility in screening settings
Shao L et al., 2020	China	Retrospective, multicenter study	575 patients	Median 70 (IQR 65–70)	Prebiopsy MRI (T2WI‐FS), needle biopsy, radical prostatectomy	Training (279), internal validation (31), external validation (178, 87)	To develop and validate a deep reinforcement learning (PCa‐GGNet) framework to predict RP Gleason grade groups	PCa‐GGNet achieved higher accuracy and reduced upgrading/downgrading vs biopsy, guiding precise treatment decisions
Fay et al., 2025	USA (Cleveland Clinic)	Retrospective, single‐institution, independent blinded validation of an AI histologic classifier (PATHOMIQ_PRAD) in RP patients with Decipher testing	344 included (of 857 screened; 486 WSIs available)	Mean age at treatment: 63 (low‐risk) vs 66 (high‐risk)	H&E whole‐slide images from biopsy or RP; RP cohort without adjuvant therapy; Decipher genomic test available	PATHOMIQ_PRAD risk: low vs high using prespecified cutoffs (0.45 for BCR, 0.55 for DM).	Validate clinical utility of PATHOMIQ_PRAD (associations with BCRFS, MFS, PFS)	High scores associated with worse BCRFS/MFS/PFS; AI histopathology adds prognostic value; merits broader/prospective validation
Yu et al., 2025 (Annals of Medicine)	China (multicenter incl. Shenyang, Shenzhen, Zhejiang, Guangxi)	Retrospective development/validation of a biopsy H&E‐based deep learning model (MIL) to predict EPE; 8:2 train/test split; explored link with BCR	260 patients (2592 H&E slides)	Median 67 (IQR 61–72)	Biopsy H&E WSIs; DL outputs EPE risk; performance compared with MSKCC nomogram, Roach formula, Partin tables	EPE vs non‐EPE; data split: training *n* = 208, testing *n* = 52	Build a DL model to predict EPE from biopsy images and assess prognostic association (BCR).	Test AUC 0.886; outperformed clinical nomograms and associated with BCR
Lee et al., 2023 (Cancers)	Republic of Korea (Asan Medical Center and Samsung Medical Center)	Retrospective cohort; preop mpMRI (T2, ADC, CE) with DL + radiomics + clinical features; fivefold cross‐validation to predict long‐term post‐RP BCR‐free survival	437 patients undergoing RP	Median 66 (IQR 61–71)	mpMRI‐derived DL/radiomic features + clinical covariates; expert‐delineated index lesions; post‐RP outcomes	BCR (*n* ˜ 110; 25.2%) vs non‐BCR (*n* ˜ 327); fivefold CV	Combine clinical and DL imaging features to improve BCR‐free survival prediction	Combined clinical–DL model had best performance (e.g., HR ˜ 7.72), outperforming clinical‐only or DL‐only models
Tang et al., 2024 (Technology and Health Care)	China (Hangzhou, Zhejiang Univ.)	Retrospective diagnostic ML/CAD development using mpMRI; goal: early PCa detection and aggressiveness estimation with lower clinician burden	106 patients; ROI image set = 433 (202 benign, 231 malignant); training 100 images (50/50), verification 333 (152 benign/181 malignant)	NR	mpMRI ROIs from T2WI/DWI/DCE used for CAD training/testing	Benign vs malignant ROI classes; separate training and verification image sets.	Build a CAD system to detect PCa/assess aggressiveness from mpMRI	Model provided a “good assessment of prostate cancer diagnosis,” potentially reducing specialist burden
Esteva et al., 2022 (npj Digital Medicine)	Multicenter NRG/RTOG Phase III trials across hundreds of centers (North America; USA/Canada affiliations listed)	Retrospective development/validation of a multimodal AI (MMAI) using five randomized Phase III RT trials; 80/20 train/validation	7764 randomized; 5654 with histopathology; median follow‐up 11.4 years	NR (age included as model covariate, but baseline age not explicitly summarized)	Biopsy digital histopathology + clinical variables (NCCN variables + age, Gleason primary/secondary)	Five Phase III cohorts (RTOG 9202/9408/9413/9910/0126).	Personalize therapy by predicting long‐term outcomes with multimodal DL	MMAI outperformed NCCN across endpoints and enables computational prediction of treatment outcomes
Spratt et al., 2023	Multicenter (USA/Canada; NRG/RTOG Phase III trials)	Retrospective development + external validation of a digital‐pathology AI predictive model using completed randomized RT ± short‐term ADT trials; primary endpoint: distant metastasis	From 7752 eligible across 5 trials, 5727 had pretreatment slides; development *n* = 2024; external validation (RTOG 9408) *n* = 1594	Median 71 (IQR 65–74) development; 71 (66–74) validation	Pretreatment prostate histopathology slides; RT alone vs RT + 4‐month ADT	Model‐positive vs model‐negative subgroups; validation arms RT vs RT + ADT	Build/validate an AI model to predict benefit from adding short‐term ADT to RT	Significant model–treatment interaction. Model‐positive patients derived large benefit from ADT (sHR≈0.34), while model‐negative showed no significant benefit; overall ADT reduced distant metastasis (sHR≈0.64)
Schmidt et al., 2025 (BJU Int.)	USA (Stanford/Utah; multi‐institution authorship)	External validation diagnostic reader‐study of DeepDx® Prostate on radical‐prostatectomy whole‐mount tiles	150 RP specimens ⟶ 500 one‐mm^2^ tiles	NR	RP tissue WSIs; AI vs reference pathologist consensus	Tile‐level: benign vs cancer; GG ≥ 2 vs GG1/benign; grade‐group classification	Validate performance of a biopsy‐trained AI on RP whole‐mount material	Excellent agreement and accuracy: e.g., QWK ≈0.89 for GG classification; cancer detection sensitivity ≈0.997, specificity ≈0.88; supports generalizability to RP
Peng et al., 2023 (Sci Rep.)	USA (SEER registries)	Retrospective cohort; machine‐learning survival models (GBSA/RSF/Extra Trees) vs Cox for node‐positive PCa overall survival	3280 LNI cases (2000–2019); train 2624/validation 656	NR (age used as model covariate/categories)	Registry variables (age, stage, PSA, GS, # of positive nodes, RP/RT indicators)	Train vs validation; ML algorithms vs Cox	Create an accessible ML tool to predict OS in node‐positive PCa	ML models showed slightly higher discrimination than Cox (mean time‐dependent AUC ∼0.78 vs 0.77) with good calibration; web tool provided
Tolkach et al., 2023 (npj Precis Oncol.)	Germany and Austria (multi‐institution)	Retrospective international external validation of a clinical‐grade AI for biopsy tumor detection and Gleason grading	5922 H&E sections representing 7473 cores from 423 cases across 5 sites; grading subsets *n* = 227 and *n* = 159	NR	Biopsy WSIs (multiple scanners/sites); AI vs uropathologists	Five external detection cohorts; two grading cohorts	Validate generalizability/accuracy for detection and grading on heterogeneous external data	High detection performance (sens 0.971–1.000; spec 0.875–0.976) and grading agreement comparable to experienced pathologists; supports clinical deployment
Bertelli et al., 2022 (Front Oncol.)	Italy (Florence and Pisa)	Monocentric observational; ML/DL on prebiopsy mpMRI (T2w, ADC) to predict lesion aggressiveness; compared across PI‐RADS v2.0/v2.1 cohorts	112 patients; 132 peripheral‐zone lesions (split across PI‐RADS v2.0/v2.1 cohorts)	NR	Prebiopsy mpMRI (T2w, ADC); pathology as reference for aggressiveness (low‐ vs high‐grade)	PI‐RADS v2.0 vs v2.1 cohorts; ML/DL vs baseline PI‐RADS assessment	Test whether ML/DL improves aggressive‐lesion prediction beyond PI‐RADS	ML/DL achieved good diagnostic performance; T2w + ADC combinations and DL architectures outperformed PI‐RADS alone for aggressiveness prediction
Bulten et al., 2022—PANDA challenge (Nat Med)	International (NL, SE; external US/EU)	Global challenge with independent internal and external validation of Gleason grading AI on biopsy WSIs	12,625 WSIs from 6 sites; 10,616 development; 545 internal validation; 1071 external validation (US 741; EU 330)	NR	Prostate biopsy H&E WSIs	Multiple independently reproduced/top submissions; internal vs external validation; algorithms vs expert uropathologists	Independently evaluate whether Gleason‐grading AIs generalize across labs/scanners/continents	Diverse algorithms achieved pathologist‐level agreement on external sets (QWK ≈ 0.86–0.87), supporting prospective clinical trials
Singhal et al., 2022 (Sci Rep)	India (clinical); training and tests also include NL/SE sets	Retrospective DL system for biopsy diagnosis and grading with active learning; multicenter external and blind validation	6670 WSI total; internal test 425; external Radboud 1201; blind Karolinska 1303	NR	Core‐needle biopsy H&E WSIs	Internal vs two external cohorts	Build a domain‐agnostic DL system for cancer detection and Gleason grade‐grouping	Accuracy/*κ* (internal 89.4%/0.92); external Radboud 85.3%/0.96; external Karolinska 83.1%/0.93 — robust cross‐site generalization
Castillo et al., 2022 (Cancers)	Netherlands (Erasmus MC) with external cohorts	Validation study comparing DL vs radiomics for csPCa on mpMRI across multiple external cohorts	644 patients (4 cohorts): train 271; three unseen test cohorts 374	NR	mpMRI (T2, DWI/ADC, DCE); pathology reference	Training cohort vs three independent test cohorts	Compare DL and radiomics for csPCa classification across centers/scanners	Radiomics outperformed DL on all external tests (AUC 0.88/0.91/0.65 vs 0.70/0.73/0.44), highlighting generalizability of radiomics
Chen et al., 2024 (Urology)	China (multicenter)	ML‐based nomograms for initial staging (LPC vs APC; LAPC vs mPCa) with external validation	Train/test 362 (single center); external validation 136 (another hospital)	NR	Clinicopathologic + biomarkers (e.g., PSA, GS, MTD, ALP, RNF41)	LPC vs APC; within APC: LAPC vs mPCa	Construct and validate nomograms to predict progression stage at diagnosis	LR models best; AUCs (train/test/ext): APC 0.848/0.814/0.810; mPCa 0.940/0.913/0.910; good calibration and DCA net benefit
Bao et al., 2024 (Insights Imaging)	China (4 tertiary centers)	Retrospective multicenter radiomics for csPCa with internal and external testing; assesses added value over PI‐RADS	1616 patients (4 centers)	NR	mpMRI radiomics; PI‐RADS by junior/senior/expert readers	Training + internal test; two external tests; PI‐RADS vs adjusted_PI‐RADS	Predict csPCa and explore correlation with ISUP; evaluate assistance to PI‐RADS	Random‐forest radiomics AUCs 0.874/0.876/0.893; improved adjusted_PI‐RADS specificity; Rad‐score positively correlates with ISUP (*r* > 0.60)
Pantanowitz et al., 2020 (Lancet Digit Health)	Israel (development/deployment) with external validation (USA/South Africa)	Blinded external validation and real‐world deployment of a CNB AI algorithm (detection, grading, GP5, PNI, tumor %)	Internal test 2501 slides; external 100 cases/1627 slides; deployment 941 cases/11,429 slides	NR	Core‐needle biopsy H&E WSIs; Philips/Aperio scanners	Internal vs external; routine deployment as “second read”	Validate accuracy and demonstrate clinical deployment feasibility	External AUCs: cancer 0.991; high‐grade vs low 0.941; GP5 0.971; PNI 0.957; strong tumor‐% correlation (*r* = 0.882); flagged a missed cancer in practice

### 3.3. Comparative Performance of AI vs Conventional NCCN Methods

Most studies reported superior discrimination with AI models compared to NCCN‐based approaches:

Imaging‐based AI, including MRI‐ and PET‐based radiomics models, consistently outperformed NCCN categories or conventional clinicopathological models in predicting adverse pathology, extracapsular extension, pelvic lymph‐node metastasis, persistent PSA, and biochemical recurrence, with reported AUCs generally ranging from 0.85 to 0.95 compared with approximately 0.70–0.80 for conventional approaches [2, 12–19]. PSMA PET/CT‐based radiomics and multimodal deep learning models showed particularly strong performance for predicting pelvic lymph‐node involvement and persistent PSA after radical prostatectomy, with AUCs reported around 0.89–0.93 [13, 15].

Digital pathology AI, including deep learning applied to whole‐slide images and nondestructive 3D histopathology, improved prediction of biochemical recurrence‐free survival, metastatic disease, and overall survival [4, 20–31]. AI‐based Gleason grading achieved pathologist‐level concordance, with reported quadratic weighted kappa values approximately ranging from 0.86 to 0.93 across validation cohorts [23, 27–30]. Several studies showed that AI‐based grading or histologic classifiers were equivalent to, or superior to, expert pathologist assessment or conventional clinicopathological risk tools in predicting long‐term outcomes [21, 23, 25, 27].

PSA‐based ML models surpassed traditional PSA, PSA derivatives, and PSA velocity in predicting prostate cancer or clinically significant prostate cancer, with Perera et al. reporting improved prediction of clinically significant disease compared with PSA and PSA velocity and Chen et al. reporting an AUC of 0.918 for a multivariable ML model compared with PSA alone [32–34]. In the multicenter PSA kinetics study by Perera et al., the ML model achieved an AUC of 0.886 compared with 0.807 for PSA and 0.627 for PSA velocity [32].

Multimodal AI models combining imaging, digital pathology, and clinical variables showed the greatest performance gains, with reported AUCs often exceeding 0.85–0.95 and generally outperforming conventional clinicopathological tools or NCCN‐based stratification [1, 3, 4, 13, 20, 24, 35, 37–39]. In particular, the multimodal AI model developed and validated using NRG Oncology Phase III randomized trials reclassified 42% of patients compared with NCCN risk groups, supporting its potential value for more individualized risk assignment [1].

Treatment selection models demonstrated the ability to identify patients who may benefit from specific therapies [24, 38, 40]. For example, Armstrong et al. validated a digital pathology‐based multimodal AI biomarker in RTOG 9202 and showed that biomarker‐positive patients derived significant benefit from long‐term androgen deprivation therapy, with a subdistribution hazard ratio of 0.55, while approximately one‐third of patients were biomarker‐negative and could potentially avoid extended androgen deprivation therapy [24].

ML models for pathologic staging, including prediction of organ‐confined disease, extracapsular extension, seminal vesicle invasion, and lymph‐node involvement, achieved higher accuracy than existing nomograms or conventional clinicopathological models in several studies, with some models reporting AUCs greater than 0.92 [13, 14, 26, 41, 42].

Detailed comparative outcomes are summarized in Table 2, which contrasts AI‐based approaches with conventional NCCN stratification.

**TABLE 2:** Comparative performance of AI versus conventional NCCN methods.

Domain	AI performance	Conventional NCCN performance
Imaging (MRI, PET radiomics)	AUC 0.85–0.95; better prediction of adverse pathology, lymph‐node metastasis, persistent PSA and recurrence	AUC 0.70–0.80
Digital pathology	Improved recurrence‐free survival, metastatic disease and overall survival prediction; pathologist‐level concordance (*κ* ≈0.86–0.93); accurate reclassification	Limited granularity in risk groups
PSA‐based models	Outperformed PSA velocity; better clinically significant PCa prediction (AUC > 0.88)	PSA and PSA velocity less predictive
Multimodal AI	Highest gains when combining imaging + pathology + clinical (AUC > 0.90); reclassified 30%–42% of patients	Conventional nomograms AUC ∼0.70–0.80
Treatment selection	Identified subgroups benefiting from specific therapies (sHR ≈0.34–0.55); spared ∼1/3 from unnecessary treatment	Limited ability to personalize therapy
Pathologic staging	ML models for OC/ECE/SVI/LNI achieved AUCs > 0.92	Existing nomograms lower accuracy

### 3.4. Calibration, Validation, and Clinical Utility

While discrimination was consistently strong, fewer studies reported calibration metrics or external validation. Decision‐curve analysis, when presented, generally supported the improved net clinical benefit of AI over conventional risk stratification. However, only a minority of studies validated models in independent cohorts or across multiple institutions, limiting generalizability.

Notable exceptions included the following:

• Large multicenter international validations with thousands of patients across multiple countries and scanner/laboratory platforms
• External validation studies demonstrating robust performance across different continents (North America, Europe, and Asia)
• Studies showing maintained performance despite differences in scanners, staining protocols, and clinical workflows
• Real‐world deployment studies demonstrating clinical utility in routine practice
• Many studies incorporating decision‐curve analysis demonstrated net clinical benefit across clinically relevant threshold probabilities, supporting potential clinical implementation.

### 3.5. Quality Assessment

**FIGURE 2:**
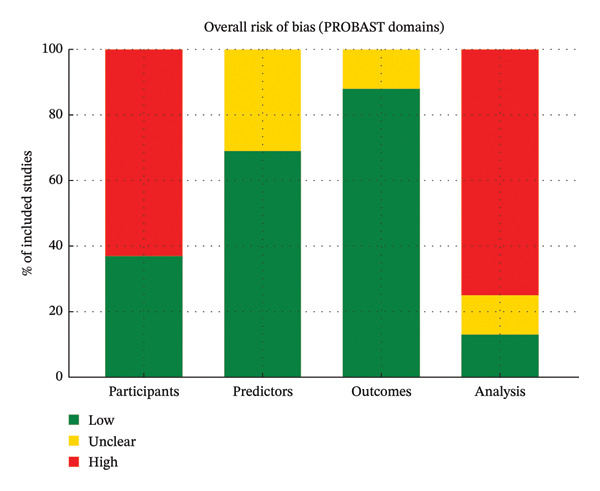
Overall risk of bias (RoB) of included studies assessed with PROBAST. This figure presents the risk of bias assessment for the 43 included studies using the PROBAST tool across four domains: participants, predictors, outcomes, and analysis. The assessment reveals mixed methodological quality, with higher risk frequently observed in the analysis domain (limited external validation and inadequate calibration reporting) and the participants domain (selection bias in single‐center retrospective studies). The predictors and outcomes domains generally showed lower risk, particularly in studies with standardized protocols and objectively defined endpoints.

The risk of bias (RoB) assessment using PROBAST (Figure 2) revealed the following:

• Participants’ domain: high risk in several retrospective cohorts due to potential selection bias, particularly in single‐center studies where patient recruitment strategies were not clearly reported. Multicenter prospective studies and randomized trial reanalyzes generally showed low risk.
• Predictors’ domain: mostly low to moderate risk; however, some studies inadequately reported feature selection, handling of missing data, or harmonization of imaging parameters across institutions. Studies with standardized imaging protocols and well‐documented preprocessing pipelines showed lower risk.
• Outcomes’ domain: generally low risk when outcomes such as biochemical recurrence, adverse pathology, metastatic disease, or overall survival were objectively defined and validated according to consensus definitions. A minority of studies relied on surrogate endpoints without robust justification.
• Analysis domain: high or unclear risk was frequent in studies that lacked external validation, failed to present calibration plots, or omitted decision‐curve analyses, limiting assessment of generalizability and real‐world clinical utility. Studies that adhered to TRIPOD‐AI guidelines showed greater transparency in reporting.

Overall, methodological quality across the included studies was mixed. While most AI‐based models demonstrated stronger discrimination than conventional NCCN risk stratification, deficiencies in external validation, calibration, and transparent reporting reduced confidence in reproducibility. Adherence to TRIPOD‐AI and the emerging PROBAST + AI guidance^8^ is strongly needed to ensure transparency, comparability, and replicability.

## 4. Discussion

The present systematic review demonstrates that AI‐based approaches consistently outperform conventional NCCN risk stratification in predicting outcomes for localized prostate cancer [1, 2, 4, 13, 15, 16, 22–24, 26–29, 35, 37–41, 43–46]. Across the 43 included studies, AI models showed higher discrimination for clinically meaningful endpoints, including adverse pathology, extracapsular extension, biochemical recurrence, metastatic disease, and overall survival [1, 2, 4, 12, 13, 15–18, 20–24, 26–30, 32, 34, 37–42, 45–48].

Imaging‐based AI, particularly MRI and PET radiomics, frequently outperformed NCCN categories or their components [2, 12, 13, 15, 16, 22, 23, 26, 27, 40, 41, 43, 47, 49, 50]. PSMA PET/CT‐based multimodal deep learning models achieved particularly impressive results, with AUCs approaching 0.89–0.93 for predicting lymph‐node metastases and persistent PSA after radical prostatectomy [15, 22, 23, 41]. Digital pathology models using deep learning on 2D whole‐slide images or nondestructive 3D pathology improved prognostication and enabled more accurate reclassification beyond standard NCCN groupings [4, 13, 17, 18, 20, 21, 24, 27–30, 37–39, 42, 44–46]. These models demonstrated pathologist‐level performance across diverse international cohorts, different scanners, and varying laboratory protocols, suggesting robust generalizability [17, 18, 28–30, 42, 46].

Multimodal AI that integrates radiological, pathological, and clinical variables yielded the largest performance gains, with AUCs often > 0.85–0.95 versus ∼0.70–0.80 for conventional approaches [1, 4, 20, 24, 35, 37, 49]. These findings suggest that AI can refine NCCN risk stratification, supporting more individualized treatment selection and potentially reducing under‐ or overtreatment [1, 4, 22, 24, 35, 37]. Importantly, several studies demonstrated that AI biomarkers could identify patients who would derive significant benefit from specific therapies (e.g., long‐term ADT) while sparing others from potentially unnecessary treatment‐related toxicity [13, 37].

Despite these promising results, several limitations temper immediate clinical adoption. External validation was infrequently reported in earlier studies; many relied on internal cross‐validation within single‐center retrospective cohorts, limiting generalizability [2, 43, 47, 49–51]. However, more recent large‐scale international multicenter validations have begun to address this gap, demonstrating robust performance across continents and diverse clinical settings [13, 17, 18, 20, 28–30, 37, 42, 46, 52–54]. Calibration was inconsistently presented, hindering assessment of absolute risk accuracy in real‐world settings [1, 2, 4, 43, 49]. Importantly, among studies that reported both discrimination and calibration, there was no consistent evidence of substantial reduction in AUC following calibration procedures. However, quantitative synthesis of AUC changes was not feasible because calibration results were reported heterogeneously, and discrimination metrics before and after recalibration were rarely presented. Consequently, the primary limitation identified relates to incomplete reporting of calibration rather than demonstrated deterioration in model discrimination. Heterogeneity in AI methods (e.g., radiomics feature sets and network architectures), outcomes, and reporting precluded meta‐analysis and limited cross‐study comparability [1, 2, 4, 20, 43, 47, 49–51]. Additionally, the predominance of retrospective designs in many earlier studies increases the RoB, particularly in the domains of participant selection and analysis [20, 47, 49–51]. Reproducibility remains an ongoing challenge, as model development details (feature handling, thresholds, and decision rules) were variably reported; adherence to TRIPOD‐AI and PROBAST + AI guidance [8, 10] is needed to improve transparency and replicability.

It is also important to recognize that limitations related to ethnic and geographic representation are not unique to AI models. NCCN risk stratification itself has historically been derived largely from cohorts in North America and Western Europe. Consequently, both conventional risk classification systems and emerging AI models require validation in more diverse populations to ensure generalizability, equity, and broad clinical applicability.

### 4.1. Clinical Implications

The findings of this review suggest that AI‐based approaches have the potential to refine NCCN risk stratification by improving the prediction of clinically meaningful outcomes, including adverse pathology, biochemical recurrence, metastatic disease, and overall survival. Incorporating multimodal AI into clinical workflows could enhance individualized treatment decisions, reduce under‐ and overtreatment, and support evidence‐based patient counseling [1, 4, 13, 20, 22, 24, 35, 37]. Real‐world deployment studies have begun to demonstrate feasibility and clinical utility in routine practice, including the identification of missed cancers and support for treatment selection [37, 42]. However, until broader prospective multi‐institutional validation is available, AI tools should be considered as adjuncts rather than replacements for conventional NCCN risk stratification.

### 4.2. Study Limitations

This systematic review has several limitations that warrant acknowledgment and should be considered when interpreting the findings.

First, heterogeneity in AI methodologies across the included studies precluded quantitative meta‐analysis. The 43 studies employed diverse ML architectures (CNNs, random forests, support vector machines, and gradient boosting), varied feature extraction approaches (radiomics, deep learning, and hand‐crafted features), and different data modalities (MRI, PET/CT, digital histopathology, clinical parameters, or multimodal combinations). This methodological diversity, while reflecting the breadth of AI applications in prostate cancer risk stratification, limited our ability to pool performance estimates and conduct formal statistical comparisons.

Second, publication bias may have influenced our findings. Studies demonstrating superior AI performance are more likely to be published than those showing null or negative results, potentially leading to overestimation of AI’s advantages over conventional NCCN stratification. Although we conducted a comprehensive search across multiple databases and included gray literature screening, we cannot exclude the possibility that unpublished studies with less favorable outcomes exist. We did not formally assess publication bias using funnel plots or Egger’s test due to the narrative nature of this synthesis and the heterogeneity of the reported outcomes.

Third, the search was limited to English‐language publications, potentially excluding relevant studies published in other languages, particularly from regions with substantial AI research activity, such as China, Japan, and other non‐English‐speaking countries. This language restriction could introduce geographic and cultural bias into our findings.

Fourth, the restriction to Q1 and Q2 journals (per SCImago rankings) may have excluded high‐quality studies published in newer or more specialized journals that have not yet achieved top‐tier rankings. While this criterion was intended to ensure methodological quality, it may have inadvertently limited the comprehensiveness of our review.

Moreover, external validation was limited or absent in a substantial proportion of the included studies, particularly those published in earlier years of the review period (2020–2022). Many studies relied on internal cross‐validation or single‐center cohorts, which limit generalizability to different patient populations, healthcare settings, imaging protocols, and laboratory practices. Although several recent large‐scale multicenter international validations have addressed this gap, the overall evidence base remains dominated by single‐institution studies with geographically and demographically homogeneous populations.

Calibration and clinical utility metrics were inconsistently reported. While discrimination (AUC and C‐index) was nearly universally reported, fewer than half of the included studies presented calibration plots, calibration statistics (Hosmer–Lemeshow test, calibration slope, calibration in the large), or decision‐curve analyses. This limits our ability to assess whether AI models provide well‐calibrated absolute risk estimates or meaningful net clinical benefit at clinically relevant decision thresholds, both of which are critical for clinical implementation.

RoB varied across the included studies, as demonstrated by our PROBAST assessment. High or unclear risk was frequently observed in the analysis domain due to inadequate handling of missing data, insufficient prespecification of modeling strategies, lack of external validation, and insufficient reporting of model development details. The participants’ domain also showed high risk in several retrospective single‐center studies where selection criteria and recruitment strategies were poorly documented, raising concerns about spectrum bias and applicability to broader clinical populations.

Reporting transparency was suboptimal in many studies, particularly those published before the introduction of TRIPOD‐AI guidelines in 2024. Key details on feature preprocessing, hyperparameter tuning, model selection, handling class imbalance, and strategies to prevent overfitting were frequently omitted or inadequately described. This lack of transparency hinders reproducibility and limits other researchers and clinicians’ ability to validate or implement these models in independent settings.

Follow‐up duration and outcome definitions varied substantially across studies. Biochemical recurrence was defined using different PSA thresholds and timeframes, and censoring strategies differed, making cross‐study comparisons challenging. Studies with short follow‐up periods (< 24 months) may not have captured late recurrences or metastatic progression, potentially underestimating the accuracy of risk stratification for long‐term outcomes.

Integration with existing clinical workflows was rarely addressed. Most studies focused on model development and validation but did not evaluate implementation feasibility, computational requirements, integration with electronic health records, or impact on clinician decision‐making and patient outcomes in real‐world settings. The lack of prospective implementation studies limits our understanding of how AI‐based risk stratification would perform when deployed in routine clinical practice.

Cost‐effectiveness analyses were absent from all included studies. The additional costs associated with advanced imaging (multiparametric MRI and PSMA PET/CT), digital pathology infrastructure, computational resources, and AI software licensing were not evaluated against potential benefits such as reduced overtreatment, improved prognostic accuracy, or enhanced treatment selection. Without economic evaluation, the value proposition for healthcare systems remains unclear.

Explainability and interpretability of AI models were infrequently discussed. Most deep learning models function as “black boxes,” providing predictions without transparent reasoning. While some studies employed attention mechanisms, saliency maps, or feature importance analyses, the majority did not address how clinicians and patients would understand, trust, and act upon AI‐generated risk estimates. This lack of interpretability poses a significant barrier to clinical adoption and may limit patient acceptance.

Ethnic and geographic diversity was limited in many included studies. Cohorts were predominantly from North America, Europe, and East Asia, with underrepresentation of patients from Africa, South America, the Middle East, and South Asia. Prostate cancer epidemiology, genetics, screening practices, and treatment patterns vary across populations, raising concerns about the generalizability of AI models trained on ethnically and geographically homogeneous datasets.

NCCN risk groups were not uniformly applied across all studies. Some studies used NCCN Version 1.2020, while others employed later versions (2021–2024), which introduced refinements in risk categorization and treatment recommendations. Additionally, several studies compared AI models to individual NCCN components (PSA, grade group, and clinical T stage) rather than composite NCCN risk groups, limiting direct comparability with the guideline‐based stratification used in clinical practice.

Baseline risk distribution varied across study cohorts, with some enriched for high‐risk disease and others focusing on intermediate‐risk or active surveillance populations. This spectrum variation affects the relative performance of AI versus conventional methods and limits the generalizability of findings to unselected screening or diagnostic populations.

### 4.3. Future Directions to Address Limitations

To overcome these limitations, future research should prioritize large‐scale prospective multicenter validation studies with diverse patient populations, standardized imaging and pathology protocols, and long‐term follow‐up. Adherence to TRIPOD‐AI and PROBAST + AI reporting and methodological guidelines is essential to improve transparency, reproducibility, and risk‐of‐bias assessment. Prospective randomized controlled trials comparing AI‐guided versus conventional NCCN‐guided treatment selection are needed to establish whether improved discrimination translates into improved clinical outcomes. Implementation studies evaluating real‐world feasibility, workflow integration, cost‐effectiveness, and impact on clinician and patient decision‐making are critical for clinical translation. Finally, the development of explainable AI methods that provide interpretable and actionable insights to clinicians and patients will be essential for building trust and facilitating adoption in routine clinical practice. The development of universally applicable AI‐based risk classification systems will likely require coordinated international collaboration. Multidisciplinary consortia led by academic societies and cooperative trial networks may provide the most effective framework for establishing standardized datasets, validation protocols, and prospective clinical trials. Engagement of national health systems and regulatory authorities will also be essential to support guideline integration, real‐world validation, and sustainable clinical implementation.

Despite these limitations, this systematic review provides a comprehensive synthesis of the current evidence base and demonstrates that AI‐based approaches consistently outperform conventional NCCN risk stratification in terms of discrimination for clinically meaningful outcomes in localized prostate cancer. Several included studies also compared AI‐based models with established clinical nomograms such as CAPRA‐S and the MSKCC nomogram, and these comparisons similarly demonstrated improved discrimination or calibration in most cases. These findings support the robustness of AI‐based approaches across multiple clinical benchmarks and highlight the importance of evaluating AI models against several validated prediction tools rather than a single risk classification system. However, the identified limitations underscore the need for continued methodological rigor, transparent reporting, and robust validation before widespread clinical implementation can be recommended.

## 5. Conclusion

AI‐based models demonstrate significant potential to improve NCCN risk stratification in localized prostate cancer, consistently showing superior discrimination compared to conventional methods across diverse geographic settings, imaging platforms, and laboratory protocols. The evidence base has expanded substantially, with 43 studies now demonstrating improved prediction of adverse pathology, biochemical recurrence, metastatic disease, and overall survival. Large‐scale international validations and real‐world deployment studies support generalizability and clinical feasibility. However, the current evidence remains limited by methodological heterogeneity in earlier studies and inconsistent reporting. For widespread clinical adoption, future research must emphasize rigorous prospective multicenter validation, transparent reporting in line with TRIPOD‐AI and PROBAST + AI guidelines, standardized AI development pipelines, comprehensive calibration assessment, and robust implementation studies demonstrating added value and cost‐effectiveness beyond established NCCN approaches. Randomized trials evaluating AI‐guided treatment selection are essential to establish definitive clinical benefit.

## Funding

This systematic review received no funding.

## Conflicts of Interest

The authors declare no conflicts of interest.

## Data Availability

The data that support the findings of this study are available from the corresponding author upon reasonable request.
